# Perioperative Management for Functional Ganglioneuroma in a 2-Year-Old Child with Multiple Congenital Defects and COVID-19: A Case Report

**DOI:** 10.5152/TJAR.2022.21247

**Published:** 2022-04-01

**Authors:** Pragadeshwaran Rajendran, Habib Reazaul Karim, Vikramjit Singh, Shruti Bais

**Affiliations:** 1 Department of Anaesthesiology, Critical Care and Pain Medicine, All India Institute of Medical Sciences, Raipur, India

**Keywords:** neuronal tumors, rare diseases

## Abstract

Unlike neuroblastoma, ganglioneuroma is a rare entity in children younger than 7 years of age. Further, these tumors are mostly inactive endocrinologically with the symptoms of abdominal pain or palpable mass. Unfortunately, when functional, they mimic or occasionally coexist with pheochromocytoma. While perioperative management of pheochromocytoma has evolved, very little is known regarding the perioperative management of functioning ganglioneuroma. Hormone secretion due to surgical manipulation and anaesthesia leads to life-threatening complications. The risk further increases when associated with other congenital comorbidities. Furthermore, the corona pandemic, in other words, coronavirus disease 2019 infection, in the perioperative period is another new challenge. We report perioperative management and outcome of a functioning retroperitoneal ganglioneuroma in a 2-year-old child, having a ventricular septal defect, spina bifida occulta, and coronavirus disease 2019. The case also highlights decision-making challenges during the coronavirus disease 2019 pandemic.

## Introduction

Ganglioneuroma is a sympathetic ganglion and adrenal medulla tumor rarely found in children younger than 7 years.^1^ Patients with ganglioneuroma are rarely symptomatic and often present with non-specific clinical features like abdominal pain or a palpable mass. However, some ganglioneuroma can be endocrinologically active, it may cause symptoms mimicking pheochromocytoma or coexist with pheochromocytoma.^2^ The incidence of such functioning ganglioneuroma is, however, not known. Besides, the lack of reliable imaging modality or biochemical test, which can effectively differentiate a functioning ganglioneuroma from pheochromocytoma, warrants surgical resection for tissue diagnosis and treatment. Perioperative complications result from catecholamine release due to anaesthetic drugs or tumor manipulation that may cause life-threatening cardiovascular effects.^3^ Thus, a well-tailored anaesthetic plan is essential for the smooth conduct of anaesthesia and better patient outcome. This report presents a functioning retroperitoneal ganglioneuroma in a 2-year-old child admitted for surgical excision. We believe such a case has been rarely reported, and the decision-making challenges during the coronavirus disease 2019 (COVID-19) pandemic make it more unique. 

### Case Presentation

**Figure 1.**
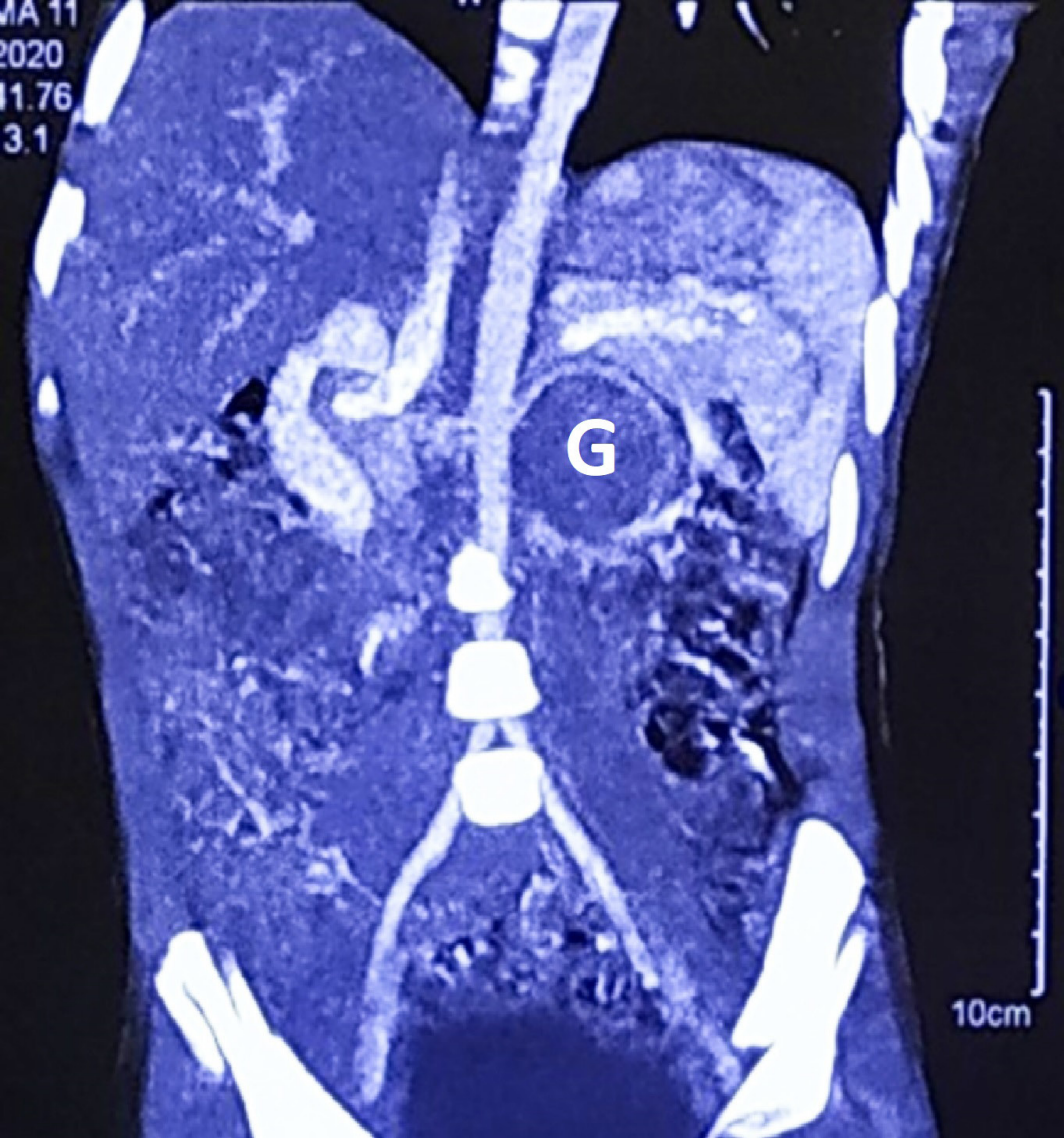
Coronal view computed tomography of abdomen showing a circular mass occupying the hilar area of the kidney, and hepatomegaly. G, ganglioneuroma.

A 2-year-old female child (11 kg) reported to our pre-anaesthesia clinic with a history of excessive sweating, hypertension, and a small ventricular septal defect diagnosed incidentally during evaluation for inguinal hernia. She was lethargic, with an heart rate (HR) 130 beats min^-1^, a blood pressure (BP) 105/72 mm Hg, and a harsh pan-systolic murmur. An electrocardiogram showed sinus rhythm and left ventricular hypertrophy. Echocardiography revealed restrictive apical muscular ventral septal defect of size 2.9 mm (<2:1 left to right shunt) with a 55 mm Hg pressure gradient, concentric left ventricular hypertrophy, normal ventricular contractility, and an ejection fraction of 56.5%. A review of imaging studies revealed a well-defined mass of 3 × 3 × 3 cm in the left para-aortic region abutting left renal hilum and calcification within the lesion, hepatomegaly spina bifida involving L5 and S1 vertebrae (Figure 1). Complete blood counts, renal, and liver function tests were within limits. Hormonal studies showed a significant rise in the levels of 24-hour urine metanephrines (57.5 µg), homovanillic acid (9.03 mg), aldosterone (31.1 ng dL^-1^), and plasma renin activity (33.96 ng mL^-1^ h^-1^).

Further, the left kidney function was only 17% in a scan. The patient received amlodipine, labetalol, and nicorandil for BP maintenance in the acceptable range except for occasional high readings. The patient underwent the planned procedure with explained high-risk consent. Her vitals were at the preoperative area: HR 114 beats min^-1^; respiratory rate 24 breaths min^-1^; temperature 38.1°C; BP 104/65 mm Hg; and room air saturation 98%. No cough, dyspnea, or flu-like symptoms were observed; COVID-19 reverse transcription polymerase chain reaction (RT-PCR) for a sample collected 3 days prior was negative. The patient was premedicated with intravenous midazolam 0.2 mg and ketamine (5 mg) and taken to the operating room. General anaesthesia (GA) was induced with slow and titrated doses of sevoflurane, propofol, and fentanyl. Atracurium was used to facilitate tracheal intubation uneventfully, and vital signs remained acceptable during the conventional laryngoscopy. Central venous access and invasive arterial monitoring were established, and landmark-guided caudal epidural block with 0.2% ropivacaine 10 mL was given with due caution. General anaesthesia was maintained with sevoflurane, fentanyl, and atracurium with low flow anaesthesia and target MACage of 1.1 ± 0.1. Dexmedetomidine 0.3 µg kg^-1^ h^-1^ infusion without loading dose was initiated immediately after induction. Hemodynamics has maintained a range of ± 20% throughout, and the patient did not require any vasoactive agents.

The patient remained afebrile after 15 mg kg^-1^ paracetamol; bleeding was within the allowable range. Reversal with neostigmine and glycopyrrolate and tracheal extubation was also uneventful. Her second nasopharyngeal sample taken on the surgery tested positive for RT-PCR of COVID-19. However, antihypertensive medications were not possible to off but titrated to achieve mean arterial pressure ranging from 50th to 95th percentile for age with 2 drugs. Unfortunately, she had an acute abdomen after 5 days, for which she required sedation service for contrast computed tomography and GA for emergency exploratory laparotomy. No complications were noted during anaesthesia management for both the sitting and she recovered well over the next week and was discharged home. No notable pulmonary or cardiovascular, or immediate COVID-19-related complications were noted.

## Discussion

The management posed several challenges: (a) functional ganglioneuroma, (b) minor yet multiple congenital abnormalities, and (c) BP was not persistently under control. Moreover, the antihypertensive regimen was lacking an alpha-blocker. Although it would have been possible to reorder the drugs to include alpha-blocker, being a COVID-19 predominant center serving more than 400 COVID-19 inpatients, minimizing the preoperative hospital stay has remained our priority so that patient does not contact COVID-19 while hospitalized. Further, the mass was pressing the renal hilum, and the left kidney was slowly going to a non-functional state. The same factors were also considered in the context of the preoperative temperature of 38.1°C. This mildly elevated temperature was single-episode and was not associated with any other signs and symptoms of infection. It, however, prompted us to take the repeat sample for COVID-19 and increased precaution during anaesthesia. 

Nevertheless, the parents belonged to a low socio-economic background and were distressed seeing the baby’s suffering and hospital shopping. So, referring to another center or ask to attend later, or postponement till the pandemic is over. Recent joint statements of the surgeons, anaesthesiologists, and perioperative nurses on the roadmap for maintaining essential surgery and guideline also indicate the same.^4,5^ Considering all these factors and following a discussion of the patient party, surgical team, and anaesthesiologists, it was decided to proceed with the scheduled surgery. However, all the precautions to contain the healthcare-associated transmission of COVID-19 were taken, intubation was done by wearing N-95 mask, including a face shield. We, however, used surgical gowns rather than a full-body personal protective equipment kit. Further, we have not used a video-laryngoscope due to logistic issues. 

Anaesthesia in surgery for a catecholamine-secreting ganglioneuroma carries a high risk of catecholamine oversecretion-associated fatal arrhythmia, and BP instability was induced by surgery, similar to surgery for pheochromocytoma.^6^ In surgery for pheochromocytoma and paraganglioma, the Endocrine Society recommends an α-adrenergic receptor blockade as the first-line drug and preoperative treatment for at least 7-14 days before surgery.^7^ Preoperative antihypertensive therapy is recommended in patients with sustained or paroxysmal hypertension and even in normotension.^8^ Careful titration should be used with α-blockers in patients with congenital heart diseases like a ventricular septal defect as the drop in systemic vascular resistance and increase in venous return might be deleterious. As in our case, preoperative calcium channel blockers and intraoperative dexmedetomidine have also been used to achieve suitable degrees of the α-adrenergic blockade.^9^ As the functioning ganglioneuroma tumors are rare in the pediatric population, no criteria are established to ascertain the adequacy of α-adrenergic blockade preoperatively. Further, mandatory use of preoperative alpha-blocker is also questioned.^10^

Intraoperatively, catecholamine secretion secondary to drug administration, stress, or manipulation of the tumor should be taken care of. We achieved it with a slow and controlled anaesthesia induction, combining with regional anaesthesia as part of multimodal analgesia, and good communication among the perioperative team members. Anticipating immediate vasodilation of the chronically constricted vasculature with anaesthesia induction, we infused 100 mL of Ringer’s lactate during this period. The additional fluid dose was managed as per loss and basal requirement. Agents that may cause arrhythmias, release histamine (morphine), or release catecholamines (desflurane) should either be avoided or administered carefully.^3^ We have used atracurium without any clinically significant side effects. It was done as per routine practice for pediatric cases in our institute and renal involvement in the patient. Rocuronium or vecuronium causes less histamine release and can be considered in patients without hepatic and renal impairment. However, all anaesthetic drugs and techniques have been used with success.^9^ Invasive blood pressure monitoring is an absolute indication, and central venous access should be considered to administer vasoactive agents and fluid management.^3^ With proper preoperative and expectant management of BP, HR, and intravascular volume, the anaesthesiologist can minimize the intraoperative complications and reduce the risk of adverse outcomes.

### Conclusions

Perioperative management of functional ganglioneuroma can be well-done as per pheochromocytoma. The optimal condition is desirable but may not be feasible and such cases need an individualized, tailored anaesthesia management plan. Calcium channel blocker, a beta-blocker drug with additional alpha-blockade activity in the preoperative antihypertensive regimen, and intraoperative dexmedetomidine might be an alternative to alpha-blocker for such a scenario.

### Informed Consent

Informed Consent: Written informed consent was obtained from the patient’s parents who agreed to take part in the study.

### Peer-review:

Externally peer-reviewed.

### Author Contributions:

Case Management – P.R., H.M.R.K., V.S., S.B.; Data Collection – P.R., H.M.R.K., V.S., S.B.; Literature Search – P.R., H.M.R.K., V.S., S.B.; Manuscript Draft – P.R., V.S., S.B.; Manuscript Revison – V.S., S.B.; Manuscript Editing – H.M.R.K. 

### Declaration of Interest

The authors have no conflict of interest to declare.

### Funding

The authors declared that this study has received no financial support.
